# Maternal deaths among rural–urban migrants in China: a case–control study

**DOI:** 10.1186/1471-2458-14-512

**Published:** 2014-05-27

**Authors:** Jingxu Zhang, Xiaozhuang Zhang, Liqian Qiu, Ronglian Zhang, David B Hipgrave, Yan Wang, Pei Zhang, Ruyan Pang, Sufang Guo

**Affiliations:** 1 Department of Child, Adolescent and Women’s Health, School of Public Health, Peking University Health Science Center, Xueyuan Road 38, Beijing, Haidian District 100191, China; 2 Guangdong Women’s and Children Hospital, Guangyuanxi Lu 13, Yuexiu District, Guangzhou, Guangdong 510010, China; 3 Women’s Hospital, School of Medicine, Zhejiang University, Xueshi Lu 1, Hangzhou, Zhejiang 310006, China; 4 Fujian Provincial Maternity and Child Health Hospital, Daoshan Lu 18, Gulou District, Fuzhou, Fujian 350001, China; 5 UNICEF, 3 UN Plaza, New York, 10017, USA; 6 Maternal and Children Health Association of China, Wanquanhe Lu Xiaonanzhuang 400, Beijing, Haidian Diastrict 100080, China; 7 UNICEF Office for China, 12 Sanlitun Lu, Beijing, 100600, China

**Keywords:** Maternal mortality, Associations, Risk factors, Rural–urban migrant women, Systematic management, China

## Abstract

**Background:**

Disparity in maternal mortality exists between rural–urban migrant and urban resident women in China, but little research has provided evidence for related policy development. The objective of this study was to identify associations with and risks for maternal death among rural–urban migrant women in order to improve health services for migrant women and reduce maternal mortality in China.

**Methods:**

We conducted a prospective case–control study in urban areas of Guangdong, Zhejiang and Fujian provinces and Beijing municipality. In each, migrant women who died between July 1, 2010 and October 1, 2011 were identified through reports from China’s Maternal and Child Mortality Surveillance System. For each, four matched controls were selected from migrant women who delivered in local hospitals during the same period. We compared socio-demographic characteristics, health status and health service variables between cases and controls, and used bivariate and multivariate conditional logistic regression analyses to determine associations with and risk factors for maternal death.

**Results:**

109 cases and 436 controls were assessed. Family income <2000 yuan per month (OR = 4.5; 95% CI 1.7-11.7) and lack of health insurance (OR = 1.3; 95% CI 1.1-1.6) were more common amongst women who died, as were lack of antenatal care (ANC) (OR = 22.3; 95% CI 4.3-116.0) and attending ANC only 1–4 times (OR = 5.0; 95% CI 1.6-15.5). Knowledge of danger signs during delivery was less common in this group (OR = 0.3; 95% CI 0.1-0.8).

**Conclusion:**

Differences existed between migrant women who died in pregnancy and surviving controls. The identified risk factors suggest strategies for health sector and community action on reducing maternal mortality among migrant women in China. A systematic approach to maternity care for rural–urban migrant women is recommended.

## Background

China has progressed remarkably on reducing its maternal mortality ratio (MMR), which decreased from 95 per 100,000 live births in 1990 to 24.5 in 2012, a 74.2% decline [1]. However, China’s MMR decreased much less in urban than rural areas due to deaths among rural–urban migrant women [2]. A study from Shanghai showed that 79% of maternal deaths during 2000–2009 were among migrant women, when their share of the city’s population was only 25.5% in 2000 and 54.8% in 2009 [3]. Similarly, the MMR among migrant women in Beijing from 1995–2004 (51.3) was much higher than among urban residents (17.9) [4]. Maternal health is one of three major health issues among China’s rural–urban migrants [5].

Poor uptake of services by and lack of health insurance among rural–urban migrants place them at risk. China’s migrant workers consistently underuse health services in their destination cities [6,7]; fewer migrant than resident women attend antenatal care (ANC) and fewer deliver in a hospital, even in large cities which have had many years to establish services for them [8-10]. Poor health-seeking behaviour among migrant women has also been identified internationally [11]. Migrants may have no entitlement to or difficulty accessing medical care [12], or may lack knowledge of services to which they are entitled [13].

Analyses on causes of maternal death among China’s rural–urban migrants highlight the problem of poor obstetric management, and note that these deaths are mostly avoidable [8-10]. In Beijing and Shanghai postpartum haemorrhage was a much more common cause of maternal death among migrants (25.2% and 39.9% of deaths, respectively), than among resident women (14.4% and 12.9%) [4,8]. This concurs with the findings of a study in England associating substandard care with maternal death [14]. Other research suggests that different treatment and improved coordination and referral practices would have changed the outcome of pregnancy-related deaths [15].

The standard verbal autopsy tool of the World Health Organization (WHO) is not used in China’s Maternal and Child Mortality Surveillance System (MCMSS) and systematic information is generally not collected on women who survive pregnancy, so the reasons for disparate outcomes between different urban sub-populations cannot be examined directly; only inferences can be drawn from comparisons between groups. Moreover, research has not explored relationships between risk factors and maternal deaths, or provided evidence for policy development on migrant maternal healthcare in China. To fill this void, we conducted a case–control study of maternal deaths among migrant women and matched controls, and report the findings herein.

## Methods

### Location, design and participants

Rural–urban migrants from six provinces and cities (Guangdong, Zhejiang, Shanghai, Jiangsu, Beijing and Fujian) account for 68.5% of the migrating population of China. Our study was conducted in prefecture level hospitals in three of these locations, Guangdong, Zhejiang and Fujian provinces, and in Haidian District of Beijing. China’s permanent residence registry or *hukou* system has had an especially strong effect on the country’s urban growth [7]. The system registers the residential location of every citizen and divides them into agricultural and non-agricultural groups. People who live in cities hold non-agricultural *hukou*, whereas farmers and other rural residents hold agricultural *hukou*. This system denies official residency and access to essential public services for rural–urban migrants, regardless of the duration of their unofficial residence in a city.

For the purposes of this study, we classified women who had arrived from their official place of residence and lived locally for at least half a year and who did not have a local *hukou* as migrants. Maternal death was defined using the standard WHO definition [16].

A case–control design was employed to investigate associations with maternal death among migrant women. The cases and controls were collected prospective in the above four locations from July 1, 2010 to October 1, 2011. Cases were identified as migrant maternal deaths associated with singleton pregnancy, reported by the national or provincial MCMSS. Twin pregnancies were excluded because of the difficulty identifying a sufficient number of matched controls. Ectopic pregnancy and induced abortion were also excluded because of their lack of attendance at ANC, a perceived key influence on pregnancy outcome. For each case, four matched controls were selected, each a surviving rural–urban migrant woman aged within five years of the case, and who had delivered after a singleton pregnancy during the surveyed period, at the same hospital (or, for a small number of controls aged <25 or >35 years, in nearby hospitals). All cases were reviewed by the local Maternal Death Audit Committee, which includes province, prefecture, and district/county experts in maternal and perinatal health. All relevant documents, including medical charts, experts’ audit documents, and Maternal and Child Health (MCH) Report Forms were reviewed. A final cause of maternal death was assigned by consensus among the committee members in each province, using the same guidelines to assign the cause of death for each case.

Non-attendance at ANC was assumed as a critical risk factor for maternal death [15] and a sample of 120 cases and 480 controls was estimated adequate to determine a 15 percent difference in the proportion of no ANC attendance between the cases and controls, with a power of 80%, alpha = 0.05, odds ratio (OR) =2 (Power Analysis Sample Size software, version 8.0, NCSS, Utah). Five ANC visits are recommended in China.

### Variables assessed and survey administration

Ideally, pregnant women in China will register and receive an ANC card, receive follow-up reminders and attend for timely care at the local hospital.

Questionnaires were designed by the survey team to examine key personal, demographic, socio-economic, and reproductive and health status factors for each case and control. Health insurance status and physical and financial health service access were included. For each case, the respondent was the husband/partner, head of the family or another close insider for both case and control group.

The questionnaire was based partly on WHO verbal autopsy standards and took into account standard care for pregnant women in China and related social determinants [17,18]; it was pre-tested and adjusted by the survey team before finalization. Specialist doctors in the four prefecture’s MCH hospitals were trained by the principal investigator (JZ) in all four locations, and one supervisor in each was assigned to oversee administration of the questionnaires. When a new maternal death of a migrant woman occurred, a trained doctor would attend to conduct the survey after gaining consent, and four matched controls were selected according to the criteria described above. All questionnaires were reviewed carefully by the principal investigator before data entry.

### Statistical analysis

EpiData 3.0 was used to build the database with duplicate data entry used to reduce errors. Analysis was undertaken using SPSS, version 13.0 (SPSS, IBM Corp). Chi-square testing was used to compare the characteristics. Bivariate conditional logistic regression analysis was used to compare the relative frequency of association of the variables assessed among cases and controls. Demographic characteristics such as women’s age group, education level, marital status, gravidity and parity, as well as factors significantly different in bivariate analyses were included in multivariate conditional logistic regression analysis to assess independent associations with maternal death. ORs and corresponding 95% confidence intervals (CI) were used to measure association. Statistical significance was established at the p < 0.05 level (two-tailed).

### Ethical considerations

The research plan, data collection instrument and consent form were approved by the Institutional Review Board at the Peking University Health Science Centre. Interviewers were trained in consent procedures and all interviewees signed a consent form that included a description of the study, noting the possible risks and benefits, and emphasis that participation was voluntary.

## Results

A total of 135 migrant maternal deaths occurred during the study period and 109 were enrolled in our study (the number of deaths before and during delivery and after labour were 27, 8 and 74, respectively). We excluded 15 deaths managed by an unskilled birth attendant (at home or in private clinics) because all controls had delivered in hospitals. We also excluded six who declined enrolment, three with ectopic pregnancy and two twin pregnancies. A total of 436 controls were selected according to the pre-determined criteria. Among the 53.2% of maternal deaths due to obstetric causes, 25.7% were due to obstetric haemorrhage, 16.5% were due to pregnancy-related hypertension and 11.0% due to amniotic fluid embolism.

### Sociodemographic characteristics of the respondents

The characteristics of cases and controls are summarized in Table 1. The mean age of the cases was not significantly different from the controls, but the percentage of cases aged ≤19 or ≥35 years was higher than among controls. Age was thus included in multivariate analysis. Women who died were poorer educated, had higher parity, were more likely to be unmarried or divorced and their pregnancy was more likely to have been unplanned (according to China’s family planning rules). All these associations were statistically significant (p < 0.001). Given the small sample size, we did not analyze associations with maternal death by province.

**Table 1:** Demographic characteristics of the cases and controls (n (%))

**Items**	**Cases (N = 124)**	**Controls (N = 496)**	**Chi-square test**	***P *** **value**
Age group				
≤19 years old	6 (5.5)	7 (1.6)	18.6	<0.001
20-34 years old	62 (56.9)	333 (76.4)		
≥ 35 years old	41 (37.6)	96 (22.0)		
Education level^†^				
Primary school	54 (50.0)	75 (17.2)	57.9	<0.001
Junior high school	44 (40.7)	218 (50.0)		
Senior high school or above	10 (9.3)	143 (32.8)		
Marital status				
Unmarried or divorced	23 (21.1)	35 (8.0)	15.7	0.001
Married	86 (78.9)	401 (92.0)		
Gravidity^†^				
0	218 (16.7)	109 (25.0)	8.6	0.013
1-2	68 (63.0)	206 (47.2)		
≥3	22 (20.4)	121 (27.8)		
Parity^†^				
0	35 (32.4)	198 (45.4)	18.3	<0.001
1	63 (58.3)	230 (52.8)		
≥2	10 (9.3)	8 (1.8)		

†: Data missing for 1 case.

### Analysis of the risk factors among maternal death in migrants

Bivariate analysis for risk factors is shown in Table 2. The risk of the family income <2000 renminbi (RMB) per month was 9.6 times higher among cases than the controls. Lack of health insurance and unplanned pregnancy were more common among the cases than the controls, as were several factors related to women’s health awareness and service uptake: not knowing to register the pregnancy and to attend ANC, being unfamiliar with key danger signs during delivery and unaware of the recommendation to deliver in hospital. Regarding health service provision and participation, not receiving initial advice and follow-up reminders to attend ANC were more common amongst cases, as were lacking decision-making power in the household on ANC attendance and not being registered in the healthcare system. Not having any ANC during pregnancy was 27.3 times more common amongst the cases than controls. Attendance at five or more ANC visits was significantly lower among cases than controls. No difference was found between the frequency of pregnancy complications between cases and controls, but assisted vaginal and caesarean section delivery were significantly more common amongst cases of maternal death.

**Table 2:** Bivariate conditional logistic regression analysis of the proportion of cases and controls with predicted risk factors for maternal death (n (%))

**Items**	**Cases (N = 109)**	**Controls (N = 436)**	**Crude OR**	**95% CI**
Income per month <2000 RMB	47 (44.3)	46 (10.7)	9.63**	5.13-18.05
No health insurance	87 (89.7)	220 (53.1)	1.19**	1.08-1.30
No planned pregnancy	66 (60.6)	144 (33.0)	3.75**	2.33-6.04
Not knowing to register the pregnancy	69 (63.3)	120 (27.5)	5.72**	3.49-9.39
Not knowing to attend antenatal care (ANC)	38 (34.9)	31 (7.1)	11.36**	5.59-23.12
Not knowing the danger signs of delivery	64 (58.7)	53 (12.2)	11.30**	6.55-19.50
Not knowing the recommendation to deliver in hospital	30 (27.5)	9 (2.1)	22.13**	8.56-57.21
Not received reminder to register the pregnancy	73 (67.0)	176 (40.4)	3.42**	2.14-5.48
Not received follow-up reminders to attend ANC	69 (63.3)	126 (28.9)	4.88**	3.02-7.89
No decision on ANC attendance made by woman	42 (38.5)	38 (8.7)	7.33**	4.17-12.89
Not registered in healthcare system	76 (69.7)	92 (21.1)	12.06**	6.81-21.37
Times of ANC				
0	49 (45.0)	22 (5.0)	27.34**	11.65-64.17
1-4	46 (42.2)	164 (37.6)	1.24	0.79-1.95
≥ 5	14 (12.8)	25 (57.3)	0.06**	0.03-0.12
Complications during pregnancy^§^	19(23.5)	60 (18.5 )	1.39	0.75-2.57
Mode of delivery^§^				
Vaginal	34 (42.0)	216 (66.7)	0.35**	0.21-0.59
Assisted vaginal	8 (9.9)	9 (2.8)	4.05**	1.45-11.31
Cesarean section	34 (42.0)	97 (29.9)	1.73*	1.03-2.89

§: Excludes women who died before delivery, then cases = 81, controls = 324.

*: *p* < 0.05; **: *p* < 0.01.

Multivariate conditional logistic regression analysis on associations showed that compared to controls, deceased women were more likely to: have low family income (OR, 4.5; 95% CI, 1.7-11.7); lack health insurance (OR,1.3; 95% CI, 1.1-1.6); not know delivery danger signs (OR, 3.1; 95% CI, 1.3-7.7); have not attended ANC (OR, 22.3; 95% CI, 4.3-116.0) and have ANC only 1–4 times (OR, 5.0; 95% CI, 1.6-15.5) (Table 3). These ORs refer to the model including age as a variable, but do not change significantly when it was omitted from the analysis. The other variables which were significantly associated with death in bivariate analysis (Table 2) were included in an initial model but excluded in the final model.

**Table 3:** Multivariate conditional logistic regression analysis on identified associations

**Variables**^**†**^	**OR**	**95% CI**
Family income <2000 renminbi per month	4.48	1.71-11.70
No health insurance	1.32	1.10-1.58
Not knowing danger signs during delivery	3.14	1.29-7.67
Times of ANC		
1-4 attendances	5.02	1.63-15.46
0 attendances	22.25	4.27-115.97

†The variables in multivariate model were defined as family income ≥2000 renminbi (RMB) = 0, <RMB2000 = 1; health insurance yes = 0, no = 1; not knowing the danger signs of delivery yes = 1, no = 0; Times of ANC1-4 yes = 1, no = 0; Times of ANC 0 yes = 1, no = 0.

## Discussion

Our study revealed that poorer education, low income, lack of health insurance, having poor knowledge on maternity care, being unregistered in the healthcare system during pregnancy, not attending ANC and attending ANC less than five times were more common among women whose pregnancy resulted in maternal mortality than among matched rural–urban migrant women who survived pregnancy. Moreover, more than 12% of the deceased women had been managed by an unskilled birth attendant (at home or in private clinics), although this is lower than identified by surveillance data in Shanghai during 1996–2005, Fujian Province during 2001–2006, and Wenzhou during 2003–2006 [8,9,19] , suggesting that this risk factor may be declining.

Our study confirmed strong associations between poverty and lack of health insurance and maternal death, as found previously among all women [20,21]. In China, migrants frequently undertake low-paying employment and do not have funds to pay for China’s often fee-for-service health care. Lack of health insurance is also common among China’s migrant workers [22,23]. In recent years, China committed to expanding health insurance coverage to >90% of the population, and official statistics showed that 91% and 97% in urban and rural areas respectively were covered by 2011 [24]. However, limited data show, for example that coverage among migrant workers in Shenzhen was only 45% [23], and it is well known that rural migrants cannot carry the benefits of their local insurance to destination cities when they migrate. The observed very low insurance coverage among migrant pregnant women is likely to have restricted their financial access to maternity services. Although the national government has set policy goals and directions to guide development of portable or merged insurance schemes, their design and implementation have been left to local governments [25]. Our study indicates the importance of both preparing migrant women for life in their destination point (to maximise employment prospects, and appraise them of their rights and of available services) and of improving the social security system for rural–urban migrants. A small number of cities have started pilot programmes to address migrant women’s needs; for example, Shanghai has subsidized migrant maternity care in public hospitals (avoiding illegal private clinics), with good outcomes [3,26]. However, these few pilots are small progress, compared to the action required.

Non-registration in the health system and lack of adequate ANC were also more common among the women who died. A recent study in Shanghai showed that free health system registration for migrant pregnant women has greatly increased their uptake of maternity services [27]. We could not determine why the deceased women were more often unregistered or whether their residency in the surveyed areas was known to the responsible public security offices, but lack of preparation or knowledge of services was again evident as a risk factor.

Pregnancy complications were not associated with mortality in our study, different from another case–control study of maternal mortality, in India [28] and the known risks of poor obstetric management [14,15,29,30]. The difference may reflect the high proportion of cases for which the deceased women’s status on this variable was “unknown” (among 81 cases who died after delivery, data on this variable was not available for 33). Nonetheless, in contrast to the dominance of indirect causes among cases of maternal mortality in higher income countries and among higher socio-economic groups in China [3,20], more than 50% of the maternal deaths in our study were caused directly by obstetric events, indicating that the priority for migrant women remains improved basic obstetric care. Clearly delivery services for migrant women in China should be improved and conducted according to the relevant national and global guidelines on obstetric care [31-33].

According to WHO’s maternal mortality audit criteria, 44.5% of maternal deaths are caused by a lack of knowledge among pregnant women and their family members [34]. Although we did not find an association between lacking knowledge on ANC and maternal death, we did find associations between lack of knowledge on danger signs of delivery and on the recommendation to deliver in hospital. It seems logical that health education on ANC, delivery care and those danger signs should be strengthened for migrating women and their families in China’s cities.

Overall, our research suggests that a systematic approach should be taken to the management of pregnant migrant women and to improving the access to health insurance among the migrant population in general. Registration and provision of a health card; improved coverage, quality and uptake of ANC; education of migrant women and their families on pregnancy management, risks and danger signs; ensuring appropriate referral, and appropriate management of obstetric complications should all be included. These things should not differ from the standards applying to resident women. Meanwhile, it is recommended to develop a maternal death registry for all maternal/pregnancy-related deaths based on autopsy confirmation to provide quality assurance on the data produced by the present system.

### Limitations of the findings

To our knowledge, this is the first case–control study in China to explore associations with maternal death among urban migrant women. However, limitations should be acknowledged. First, we did not include women who delivered at home or in private clinics in our analysis, because we did not include such locations in our search for controls, potentially causing a selection bias. It is known that women who deliver at home or in private clinics face additional risks [9,10], but this risk factor could not be assessed. Second, being a known risk factor for maternal mortality, an attempt to match cases and controls for age was undertaken but proved difficult, as the women who died were either younger or older than women who did not die (as expected). We therefore undertook multivariate analyses that both included and excluded age as a variable, but this did not change the identified associations with maternal death. Third, the interviewees for the cases were the deceased women’s husbands or family head, who might not have accurately reflected the knowledge or situation of the women who died. It is possible that the deceased women had more knowledge than their husbands, but were unable to act on it; this emphasizes the importance of the systematic approach recommended below. Fourth, our study used a quantitative methodology, but exploring social determinants would have benefited from qualitative data to better highlight the origins of the associations identified. This may have further explained why some groups of rural–urban migrants are better able to participate in and access services than others. Fifth, the final sample size for cases was lower than the 120 originally calculated to have 80% power to determine a 15% difference in ANC non-attendance. Nonetheless, with the sample of 109 cases and 436 controls, we were still able to show in multivariate analysis that cases were 22 times as likely to have had no ANC and five times as likely to have had fewer than the recommended five visits. Finally, we did not explore deaths due to abortion, a common practice for birth control in China, and possibly an important cause of maternal mortality. However, we believe similar associations would have been identified if this group had been included.

## Conclusions

Our study provides evidence on important associations with maternal mortality among migrant women in China, and should facilitate and stimulate the development of recommendations and strategies for related health sector and community action. This should include providing health insurance for migrant workers and their families, providing similar quality ANC to urban migrant and resident women and extending systematic management of pregnancy to migrants. Collectively, these should decrease the risk of maternal mortality in China and in other emerging economies experiencing high levels of rural–urban migration and slow decline of maternal mortality in urban areas.

## Abbreviations

MMR: Maternal mortality ratio; ANC: Antenatal care; WHO: World Health Organization; MCMSS: China’s maternal and child mortality surveillance system; MCH: Maternal and child health; OR: Odds ratio; RMB: Renminbi

## Competing interests

The authors declare that they have no competing interests.

## Authors’ contributions

JZ, RP, SG, YW and DH developed the concept, contributed to data collection, analysis and interpretation of the results. XZ, LQ, and RZ participated in the study design and data collection. JZ, RP PZ and SG participated in data collection, data analysis and results interpretation. JZ wrote the first draft of the manuscript; DH and SG edited and finalized it. All authors read and approved the final manuscript.

## Pre-publication history

The pre-publication history for this paper can be accessed here:

http://www.biomedcentral.com/1471-2458/14/512/prepub

## References

[B1] United Nations Development Programme in China UNDP 2013 report - China’s progress towards the millennium development goals 2013 report 2013 Available at: http://wcm.fmprc.gov.cn/pub/ce/cgsy/eng/zgxw/P020130922717154941911.pdf (Date 24 January, 2014)

[B2] Ministry of Health WHO UNICEF UNFPA Joint review of maternal and child survival strategy 2006 Beijing: China Ministry of Health

[B3] Du L Qin M Zhang L Xu H Zhu L Trends in maternal mortality in resident vs. migrant women in Shanghai, China, 2000–2009: a register-based analysis Reprod Health Matters 2012 20 73 80 10.1016/S0968-8080(12)39608-0 22789084

[B4] Shen R Yang H Li H He F Ding H Deng X Xiao X Liu G Study on the maternal mortality ratio from 1995 to 2004 among resident and migrant women in Beijing (in Chinese) Chin J Epidemiol 2006 27 223 225 16792889

[B5] Hu X Cook S Salazar MA Internal migration and health in China Lancet 2008 372 1717 1719 10.1016/S0140-6736(08)61360-4 18930533 PMC7135200

[B6] Nielsen I Nyland C Smyth R Zhang M Zhu CJ Which rural migrants receive social insurance in Chinese cities? Evidence from Jiangsu Survey Data Global Soc Pol 2005 5 353 381 10.1177/1468018105057416

[B7] Gong P Liang S Carlton EJ Jiang Q Wu J Wang L Remais JV Urbanisation and health in China Lancet 2012 379 843 852 10.1016/S0140-6736(11)61878-3 22386037 PMC3733467

[B8] Zhu L Qin M Du L Jia W Yang Q Walker MC Wen SW Comparison of maternal mortality between migrating population and permanent residents in Shanghai, China, 1996–2005 Int J Gynecol Obstet 2009 116 401 407 10.1111/j.1471-0528.2008.01979.x 19187372

[B9] Lin J Zheng Q Chen B Chen X Chen S Wei X Li Y Analysis of Causes of maternal death in floating population (in Chinese) Str J Prev Med 2008 14 87 88

[B10] Gu Y Li Z Lu Y Analysis of maternal death in Wuxi from 2001 to 2006 (in Chinese) Matern Child Health Care China 2009 24 4822 4823

[B11] Ameh CA Van den Broek N Increased risk of maternal death among ethnic minority women in the UK Obstetrician Gynaecologist 2008 10 177 182 10.1576/toag.10.3.177.27421

[B12] Sanmartin C Ross N Experiencing difficulties accessing first-contact health services in Canada: Canadians without regular doctors and recent immigrants have difficulties accessing first-contact healthcare services. Reports of difficulties in accessing care vary by age, sex and region Health Policy 2006 1 103 109 PMC2585333 19305660

[B13] D’Souza L Garcia J Improving services for disadvantaged childbearing women Child Care Health Dev 2004 30 599 611 10.1111/j.1365-2214.2004.00471.x 15527471

[B14] Waterstone M Bewley S Wolfe C Incidence and predictors of severe obstetric morbidity: case control study Br Med J 2001 322 1089 1094 10.1136/bmj.322.7294.1089 11337436 PMC31259

[B15] Sharma BR Gupta N Forensic consideration of pregnancy-related deaths: An overview J Forensic Leg Med 2009 16 233 238 10.1016/j.jflm.2008.12.005 19481703

[B16] World Health Organization International statistical classification of diseases 2010 Geneva, Switzerland: WHO

[B17] Zhuang C Zhu H Lin F Huang Y Wu Q Cheng L Prenatal health care and pregnancy outcomes of internal migrants: a controlled trial Prog Obstet Gynecol 2007 16 425 428

[B18] World Health Organization Verbal autopsy standards: ascertaining and attributing cause of death 2007 Geneva: WHO

[B19] Xia J The analysis and strategy of maternal deaths in the migrants (in Chinese) Matern Child Health Care China 2008 23 4243 4244

[B20] Ronsmans C Graham WJ Maternal mortality: who, when, where, and why Lancet 2006 368 1189 1200 10.1016/S0140-6736(06)69380-X 17011946

[B21] Feng X Zhu J Zhang L Song L Hipgrave D Guo S Ronsmans C Guo Y Yang Q Socio-economic disparities in maternal mortality in China between 1996 and 2006 BJOG 2010 117 1527 1536 10.1111/j.1471-0528.2010.02707.x 20937073

[B22] Gao Q Yang S Li S Labor contracts and social insurance participation among migrant workers in China China Econ Rev 2012 23 1195 1205 10.1016/j.chieco.2012.09.002

[B23] Mou J Cheng J Zhang D Jiang H Lin L Griffiths SM Health care utilisation amongst Shenzhen migrant workers: does being insured make a difference? BMC Health Serv Res 2009 9 214 10.1186/1472-6963-9-214 19930580 PMC2788549

[B24] Meng Q Xu L Zhang Y Qian J Cai M Xin Y Gao J Xu K Boerma JT Barber SL Trends in access to health services and financial protection in China between 2003 and 2011: a cross-sectional study Lancet 2012 379 805 814 10.1016/S0140-6736(12)60278-5 22386034

[B25] Hipgrave D Guo S Mu Y Guo Y Yan F Scherpbier R Brixi H Chinese-style decentralization and health system reform PLoS Med 2012 9 11 e1001337 doi:10.1371/journal.pmed.1001337 10.1371/journal.pmed.1001337 23139644 PMC3491007

[B26] Zhu L Qin M Jia W Whole coverage of maternal health management and effect in Shanghai (in Chinese) J Chin Mater Child Healthc 2008 23 1321 1323

[B27] Yue T Fan X Study on the effectiveness of free registering in prenatal health care management among floating pregnant women (in Chinese) Chin J Women Child Health 2012 3 139 140

[B28] Gupta SD Khanna A Gupta R Sharma NK Sharma ND Maternal Mortality Ratio and predictors of maternal death in selected desert districts in Rajasthan a community-based survey and case–control study Women’s Health Issues 2010 20 80 85 10.1016/j.whi.2009.10.003 20123178

[B29] Hoyert DL Danel I Tully P Maternal mortality, United States and Canada, 1982–1997 Birth 2000 27 4 11 10.1046/j.1523-536x.2000.00004.x 10865554

[B30] Nannini A Weiss J Goldstein R Fogerty S Pregnancy-associated mortality at the end of the twentieth century: Massachusetts, 1990–1999 J Am Med Womens Assoc 2002 57 140 143 12146603

[B31] World Health Organization, Western Pacific Region China country profile 2011 Available at http://www.wpro.who.int/countries/chn/5CHNpro2011_finaldraft.pdf (Last checked 31 January, 2014)

[B32] World Health Organization WHO recommendations for prevention and treatment of pre-eclampsia and eclampsia 2011 Geneva: WHO 23741776

[B33] World Health Organization WHO recommendations for the prevention and treatment of postpartum haemorrhage 2012 Geneva: WHO 23586122

[B34] Ding H Zhang L Analysis of national maternal mortality rate surveillance (in Chinese) Matern Death Investig Cooperative China 1999 34 645 648 11486784

